# Diabetes and visual impairment in sub-Saharan Africa: evidence from Cameroon

**DOI:** 10.1186/s40200-015-0151-4

**Published:** 2015-04-08

**Authors:** Ahmadou M Jingi, Jobert Richie N Nansseu, Jean Jacques N Noubiap, Yannick Bilong, Augustin Ellong, Côme Ebana Mvogo

**Affiliations:** Department of Internal Medicine and Specialties, Faculty of Medicine and Biomedical Sciences, University of Yaoundé I, Yaoundé, Cameroon; Department of Public Health, Faculty of Medicine and Biomedical Sciences, Yaoundé, Cameroon; Internal Medicine Unit, Edéa Regional Hospital, PO Box 100, Edéa, Cameroon; Medical Diagnostic Center, Yaoundé, Cameroon; Department of Ophthalmology, Faculty of Medicine and Biomedical Sciences, University of Yaoundé I, Yaoundé, Cameroon

**Keywords:** Visual impairment, Diabetes, Diabetic retinopathy, Diabetic maculopathy, Cataract, Glaucoma, Cameroon, Sub-Saharan Africa

## Abstract

**Background:**

“Vision 2020 – the right to sight” is a program which purpose is to eliminate avoidable blindness by the year 2020 through the implementation of concrete action plans at the national and district levels. Accordingly, baseline data are needed for the planning, monitoring, follow-up and evaluation of this program. The present study aimed to better characterize visual impairment and blindness in Cameroonian diabetics by providing with baseline data on the prevalence and main causes of these affections.

**Methods:**

This was a hospital-based cross-sectional study, conducted from October 2004 to October 2006 at the Department of Ophthalmology of the Douala General Hospital, Cameroon. We included 407 diabetic patients who were referred from diabetes clinics for ophthalmologic evaluation. Ophthalmologic data included visual acuity, intra-ocular pressure, fundoscopy and fluorescein angiography.

**Results:**

The prevalence of blindness and poor vision were respectively 12.3% and 17.4% with regard to the worst eyes. Fifty nine (14.5%) patients were found with diabetic maculopathy, of whom 25.4% (15/59) had poor vision, and 25.4% (15/59) were blind. The prevalence of sight threatening retinopathy (severe non-proliferative and proliferative) was 17.4%. The degree of visual impairment was comparable in both diabetic types (p = 0.825), and it increased with the severity of retinopathy (p < 0.0001), as well as that of maculopathy (p <0.0001). The prevalence of glaucoma was 15% (61/407) when considering the worst eyes. The severity of visual impairment increased with the severity of glaucoma (p = 0.001). One hundred and twenty-one (29.7%) patients presented with cataract irrespective of its location or severity. Cataract was significantly associated with poor vision and blindness (p < 0.0001). Hypertensive retinopathy (4.9%), papillary ischaemia (2.7%), vaso-occlusive eye disease (2.5%), and age-related macular edema (2%) were the other potential causes of visual impairment and blindness encountered the most in our setting. Age ≥ 50 years, male sex, duration of diabetes and hypertension variously increased the risk of having glaucoma, cataract, diabetic retinopathy or maculopathy.

**Conclusion:**

Poor vision and blindness are frequent in Cameroonian diabetics, and their causes are similar to those reported by various other surveys: mainly cataract, glaucoma, diabetic retinopathy and maculopathy.

## Background

There are 161 million people with visual impairment in the world, of whom approximately 37 million are blind [1]. Cataract accounts for most cases of blindness [1-5]. Blindness due to cataract is becoming widespread in developing countries owing to the increasing lifespan, the population getting thereby more and more aged [1]. Likewise, glaucoma is another important cause of visual impairment and blindness in developing countries, specifically in blacks where it occurs at a younger age (<40 years) [6-9]. Cataract and glaucoma are, one and the other, two foremost ocular complications of diabetes besides diabetic retinopathy and maculopathy, and other potential blinding complications such as ischemic optic neuropathy, extra-ocular muscle palsy, iridocyclitis and rubeosis iridis [4]. Diabetes has reached epidemic proportions fuelled by an ageing population as well as the rapid increase of obesity, extending its greatest impact especially on developing countries’ adults [10,11]. Lack of services to tackle the disease adds to the burden of avoidable blindness in developing countries.

The slogan “Vision 2020 – the right to sight” was launched in 1999, the purpose of which was to eliminate avoidable blindness by the year 2020, through the implementation of effective and concrete action plans at the national and district levels. Accordingly, baseline data are needed for the planning, monitoring, follow-up and evaluation of vision 2020 programs. But unfortunately, there is currently no baseline data on visual impairment and blindness in Cameroonian diabetic patients, this being not uncommon in many other Sub-Sahara African Countries. The present study aimed to determine the prevalence, characteristics and causes of visual impairment and blindness among Cameroonian diabetic patients.

## Methods

### Ethics statement

This study was approved by the National Ethics Committee of Cameroon. We obtained written informed consent from all the participants.

### Study population and setting

The study was conducted from October 2004 through October 2006 in the Department of Ophthalmology of the Douala General Hospital, which has been extensively described elsewhere [12]. We included all diabetic patients who were referred from diabetes clinics for ophthalmologic evaluation during the study period. From these potential participants, we excluded those with incomplete data.

### Study procedure

After collecting sociodemographic data such as age, sex and medical past history including type and duration of diabetes, a careful ophthalmologic evaluation was performed. Presenting and best-corrected visual acuity were measured using projection charts placed at a 6 m distance from the patient. Patients who could only count their fingers at 3 m and 1 m were attributed a visual acuity of 1/20 and 1/50 respectively. Visual acuity was recorded and classified according to the International Council of Ophthalmology (ICO) classification of visual impairment and blindness [13].

Intraocular pressure was measured by Goldman applanation tonometry incorporated into the biomicroscope, after instilling in each of the two eyes one drop of a local anaesthetic (amethocaine 0.5% or oxybupricaine 0.4%), followed by the instillation of one drop of a diluted solution of sodium fluorescein 10%. The biomicroscope was used with a +90 dioptres lens (Volk). Afterwards, the pupils were dilated with mydriatic drops, consisting in a combination of tropicamide 0.5% (Mydriaticum ®) with phenylephrine HCl 10% (Neosynephrine ®), instilled 3 times at a constant interval of 5 minutes. Fundoscopy was performed 45 minutes after the last drop has been instilled.

Biomicroscopy was performed with a +90 dioptres lens. Indirect ophthalmoscopy was performed as a supplement with a 3-mirrored lens in eyes with high risk of retinal detachment, to visualise the peripheral retina. Fluorescein angiography on its own was performed with a KOWA RC –XV2 angiograph as a complement to ophthalmoscopy. Serial photographs were taken with blue light after injecting 5 ml of sodium fluorescein 10% into a good antebrachial vein, under strict asepsis.

### Measurements and definitions

We used the following definitions, in keeping with the ICO classification of visual impairment and blindness [13]:

- Normal or near normal vision: VA 3/10 – 10/10.
- Mild poor vision or type I: VA 1/10 – 3/10.
- Severe poor vision or type II: VA 1/20 – 1/10.
- Legal blindness or type III: VA 1/50 – 1/20.
- Quasi total blindness or type IV: VA < 1/50, Light perception (+).
- Total blindness or type V: No light perception.
- Cataract: Any lens opacity.
- Glaucoma: (i) Intraocular pressure > 20 mmHg with glaucomatous optic disc changes, (ii) Normal intraocular pressure with glaucomatous disc changes, (iii) Normal intraocular pressure, the patient being on long term anti-glaucomatous medication with or without glaucomatous disc changes.
- Diabetic retinopathy was classified as non-proliferative or proliferative.
- Diabetic maculopathy: Any retinal thickening in and around the macula.
- Visual impairment: Any poor vision or blindness.

### Statistical methods

Data were coded, entered and analyzed using the Statistical Package for Social Science (SPSS) version 20.0 for Windows (SPSS, Chicago, Illinois, USA). We described continuous variables using mean and standard deviation (SD) or median and interquartile range (IQR), and categorical variables using their frequency and percentage. The chi-square test or its equivalent was used to compare categorical variables. We calculated odds ratios (OR) with both univariate and multivariate logistic regression analyses while adjusting for confounders, in order to seek for factors influencing the occurrence of the different causes of visual impairment. A *p* value less than 0.05 was considered statistically significant.

## Results

On the whole, 407 diabetic subjects were retained, of whom 170 (41.8%) were women and 358 (88%) were type 2 diabetics (Table 1). Their ages ranged from 13 to 90 years, with a mean of 54.2 years (SD = 11.2), and they have been diagnosed with diabetes for a median duration of 5 years (IQR, 0.3–11) (Table 1).

**Table 1:** **Background characteristics of study population (N = 407)**

**Characteristic**	**Frequency (%) or Mean (SD), Median (IQR)**
**Age (years)**	
Overall	54.2 (11.2)
Female	55.7 (12.3)
Male	53.2 (10.3)
**Gender**	
Male	237 (58.2)
Female	170 (41.8)
**Type of Diabetes**	
Type 1	49 (12)
Type 2	358 (88)
**Duration of diabetes (years)**	
Overall	5 (0.3–11)
Type 1 diabetes	1 (0–11)
Type 2 diabetes	5 (0.8–11)
**Hypertension**	
Absent	246 (40.4)
Present	161 (39.6)

As shown in Table 2, the prevalence of poor vision and blindness was respectively 11.8% and 14.0% for the right eyes, and 14.2% and 12.3% for the left ones. But when considering the most affected eye, the prevalence rates of poor vision and blindness were 12.3% and 17.4 respectively. Table 3 depicts the prevalence rates of diabetic retinopathy and maculopathy with regard to visual acuity. Fifty nine (14.5%) patients were found with diabetic maculopathy, of whom 25.4% (15/59) had poor vision (types I and II), and 25.4% (15/59) were blind (types III, IV and V) with respect to the right eyes, these findings being similar in the case of the left eyes. As shown in Table 3, 60 (14.7%) patients presented with proliferative retinopathy, 21.7% (13/60) of whom had poor vision (types I and II) whilst 41.7% (25/60) were blind (types III, IV and V). The prevalence of sight threatening retinopathy (severe non-proliferative and proliferative) was 17.4%. The degree of visual impairment was comparable in both diabetic types (p = 0.825), and it increased with the severity of retinopathy (p < 0.0001), as well as that of maculopathy (p <0.0001).

**Table 2:** **Distribution of visual impairment**

**Category of vision**	**Visual acuity**	**Right eye**	**Left eye**
		**n (%)**	**n (%)**
Normal or near-normal vision	3/10–10/10	302 (74.2)	299 (73.5)
Mild poor vision (type I)	1/10–3/10	46 (11.3)	48 (11.8)
Severe poor vision (type II)	1/20–1/10	11 (2.7)	10 (2.4)
Legal blindness (type III)	1/50–1/20	11 (2.7)	13 (3.2)
Quasi-total blindness (type IV) and total blindness (type V)	<1/50, LP*(+), LP*(−)	37 (9.1)	37 (9.1)

*LP: Light perception.

**Table 3:** **Retinopathy and maculopathy with respect to visual acuity**

	**Retinopathy**			**Maculopathy**	
	**n (%)**			**n (%)**	
**Category of vision**	**None (n = 243)**	**Non-proliferative (n = 104)**	**Proliferative (n = 60)**	**Absent (n = 348)**	**Present (n = 59)**
Normal or near-normal vision	208 (85.6)	72 (69.2)	23 (38.3)	274 (78.7)	29 (49.1)
Mild poor vision	18 (7.4)	21 (20.2)	7 (11.7)	36 (10.3)	10 (17.0)
Severe poor vision	3 (1.2)	3 (2.9)	5 (8.3)	6 (1.7)	5 (8.5)
Legal blindness	7 (2.9)	1 (1.0)	3 (5)	8 (2.3)	3 (5.1)
Quasi-total blindness and total blindness	7 (2.9)	7 (6.7)	22 (36.7)	24 (6.9)	12 (20.3)
***p*** **value**	**<0.0001**			**<0.0001**	

On its own, the prevalence of glaucoma was 14.7% (60/407) with concern to the right eyes (ocular pressure > 20 mmHg), with 16.7% (10/60) of these patients having a poor vision (types I and II), and 23.3% (14/60) of them being blind (types III, IV and V) (Table 4). Neovascular glaucoma accounted for 6.7% (4/60) of all glaucomas, representing about 1% of all patients. The severity of visual impairment increased with the severity of glaucoma (p = 0.001).

**Table 4:** **Intraocular pressure and visual acuity**

	**Right ocular pressure**			**Left ocular pressure**		
	**n (%)**			**n (%)**		
**Category of vision**	**<20 mmHg**	**21–30 mmHg**	**>30 mmHg**	**<20 mmHg**	**21–30 mmHg**	**>30 mmHg**
	**(n = 347)**	**(n = 48)**	**(n = 12)**	**(n = 346)**	**(n = 48)**	**(n = 12)**
Normal or near-normal vision	267 (76.9)	30 (62.5)	6 (50.0)	267 (77.2)	31 (63.3)	5 (41.7)
Mild poor vision	38 (11.0)	7 (14.6)	1 (8.3)	40 (11.6)	6 (12.2)	0 (0)
Severe poor vision	9 (2.6)	2 (4.2)	0 (0)	8 (2.3)	2 (4.1)	1 (8.3)
Legal blindness	10 (2.9)	1 (2.1)	0 (0)	8 (2.3)	2 (4.1)	1 (8.3)
Quasi-total blindness and total blindness	23 (6.6)	8 (16.6)	5 (41.7)	23 (6.6)	8 (16.3)	5 (41.7)
***p*** **value**	**0.002**			**0.001**		

Twenty nine point seven per cent (121/407) of participants presented with cataract irrespective of its location or severity (Table 5). When considering the right eye as the reference, 22.3% (27/121) of patients exhibiting cataract had poor vision (types I and II) while 21.5% (26/121) were blind (types III, IV and V). Cataract was significantly associated with poor vision and blindness (p < 0.0001).

**Table 5:** **Cataract and visual acuity**

	**Right ocular cataract**		**Left ocular cataract**	
	**n (%)**		**n (%)**	
**Category of vision**	**Absent**	**Present**	**Absent**	**Present**
	**(n = 286)**	**(n = 121)**	**(n = 286)**	**(n = 121)**
Normal or near-normal vision	235 (82.2)	68 (62.5)	242 (84.6)	61 (50.4)
Mild poor vision	26 (9.1)	20 (14.6)	16 (5.6)	30 (24.8)
Severe poor vision	4 (1.4)	7 (4.2)	4 (1.4)	7 (5.8)
Legal blindness	5 (1.7)	6 (2.1)	7 (2.5)	4 (3.3)
Quasi-total blindness and total blindness	16 (5.6)	20 (16.6)	17 (5.9)	19 (15.7)
***p*** **value**	**<0.0001**		**<0.0001**	

Table 6 presents other fundoscopic and angiographic findings that may result in poor vision and blindness. Hypertensive retinopathy (4.9%), papillary ischaemia (2.7%), vaso-occlusive eye disease (2.5%), and age-related macular oedema (2%) were other potential causes of visual impairment and blindness mostly encountered in our setting.

**Table 6:** **Other causes of visual impairment**

**Cause**	**Frequency (%)**
Other maculopathies	2 (0.5)
Drusen	2 (0.5)
Pigment retinopathy	3 (0.7)
Papillary ischaemia	11 (2.7)
Vaso-oclusive eye disease	10 (2.5)
Age-related macular oedema	8 (2.0)
Central serous retinopathy	1 (0.3)
Vitreous degeneration	3 (0.7)
Hypertensive retinopathy	20 (4.9)

Correlates of causes of visual impairment are presented in Table 7 and Table 8. In multivariate analysis, an age greater than 50 years was associated with the risk of having a severely impaired vision (at least severe poor vision) (OR: 6.03, 95% CI 2.98–12.20; *p* < 0.001), a cataract (OR: 3.44, 95% CI 2.21–5.38; *p* < 0.001) and a diabetic retinopathy (OR: 1.79, 95% CI 1.20–2.67; *p* = 0.004). Females were less likely to have a diabetic retinopathy (OR: 0.59, 95% CI 0.42–0.84; *p* = 0.003) and a maculopathy (OR: 0.53, 95% CI 0.35–0.83; *p* = 0.005). A duration of diabetes greater than 10 years was associated with the presence of glaucoma (OR: 1.76, 95% CI 1.16–2.68; *p* = 0.008), cataract (OR: 1.42, 95% CI 1.02–1.99; *p* = 0.035), diabetic retinopathy (OR: 2.94, 95% CI 2.06–4.20; *p* < 0.001) and maculopathy (OR: 3.96, 95% CI 2.48–6.35; *p* < 0.001). Hypertensive patients were at higher risk of having glaucoma (OR: 1.51, 95% CI 0.99–2.28; *p* = 0.049), cataract (OR: 1.51, 95% CI 1.09–2.10; *p* = 0.013) and diabetic retinopathy (OR: 1.86, 95% CI 1.32–2.62; *p* < 0.001).

**Table 7:** **Unadjusted correlates of causes of visual impairment**

**Variable**	**Outcome**	**Unadjusted odds ratio**	**95% Confidence Interval**	***p*** **value**
**Age**				
Less than 50 years old		1		
50 years old and above	Severely impaired vision^*^	6.84	3.41 – 13.73	<0.001
	Presence of glaucoma	1.74	1.10 – 2.75	0.017
	Presence of cataract	4.29	2.85 – 6.45	<0.001
	Presence of diabetic retinopathy	2.30	1.58 – 3.36	<0.001
	Presence of maculopathy	1.43	0.92 – 2.23	0.115
**Sex**				
Male		1		
Female	Severely impaired vision	1.01	0.69 – 1.51	0.939
	Presence of glaucoma	1.02	0.69 – 1.51	0.909
	Presence of cataract	1.24	0.91 – 1.68	0.166
	Presence of diabetic retinopathy	0.66	0.48 – 0.92	0.013
	Presence of maculopathy	0.57	0.37 – 0.86	0.008
**Type of diabetes**				
Type 1		1		
Type 2	Severely impaired vision	2.19	0.86 – 5.59	0.099
	Presence of glaucoma	1.81	0.76 – 4.29	0.178
	Presence of cataract	2.68	1.35 – 5.36	0.005
	Presence of diabetic retinopathy	0.64	0.37 – 1.11	0.107
	Presence of maculopathy	0.62	0.33 – 1.15	0.131
**Duration of diabetes**				
Less than 10 years		1		
10 years and above	Severely impaired vision	1.97	1.31 – 2.94	0.001
	Presence of glaucoma	2.03	1.36 – 3.04	0.001
	Presence of cataract	2.03	1.49 – 2.76	<0.001
	Presence of diabetic retinopathy	3.61	2.57 – 5.09	<0.001
	Presence of maculopathy	4.16	2.62 – 6.59	<0.001
**Presence of Hypertension**				
No		1		
Yes	Severely impaired vision	1.64	1.11 – 2.43	0.014
	Presence of glaucoma	1.77	1.19 – 2.65	0.005
	Presence of cataract	1.89	1.39 – 2.57	<0.001
	Presence of diabetic retinopathy	2.29	1.66 – 3.17	<0.001
	Presence of maculopathy	1.67	1.12 – 2.50	0.012

^*^Severely impaired vision: visual acuity <1/10.

**Table 8:** **Adjusted correlates of causes of visual impairment**

**Variable**	**Outcome**	**Adjusted odds ratio**	**95% Confidence Interval**	***p*** **value**
**Age**				
Less than 50 years old		1		
50 years old and above	Severely impaired vision^*^	6.03	2.98 – 12.20	<0.001
	Presence of glaucoma	1.47	0.92 – 2.36	0.109
	Presence of cataract	3.45	2.21 – 5.38	<0.001
	Presence of diabetic retinopathy	1.79	1.20 – 2.67	0.004
**Sex**				
Male		1		
Female	Presence of diabetic retinopathy	0.59	0.42 – 0.84	0.003
	Presence of maculopathy	0.54	0.35 – 0.83	0.005
**Type of diabetes**				
Type 1		1		
Type 2	Presence of cataract	1.15	0.53 – 2.49	0.725
**Duration of diabetes**				
Less than 10 years		1		
10 years and above	Severely impaired vision	1.45	0.95 – 2.22	0.085
	Presence of glaucoma	1.76	1.16 – 2.68	0.008
	Presence of cataract	1.43	1.02 – 1.99	0.035
	Presence of diabetic retinopathy	2.94	2.06 – 4.20	<0.001
	Presence of maculopathy	3.97	2.48 – 6.36	<0.001
**Presence of Hypertension**				
No		1		
Yes	Severely impaired vision	1.31	0.87 – 1.98	0.194
	Presence of glaucoma	1.51	0.99 – 2.28	0.049
	Presence of cataract	1.51	1.09 – 2.10	0.013
	Presence of diabetic retinopathy	1.86	1.32 – 2.62	<0.001
	Presence of maculopathy	1.33	0.87 – 2.03	0.185

^*^Severely impaired vision: visual acuity <1/10.

## Discussion

The present study figures out that the prevalence of poor vision (types I and II, VA 1/20 – 3/10) was 17.4% while that of blindness (types III, IV, and V, VA < 1/20) was 12.3% in the worst eyes. Unsurprisingly, the dominating causes of poor vision and blindness were, from the most to the less encountered: cataract (29.7%), sight threatening retinopathy (17.4%), glaucoma (15%).and maculopathy (14.5%). These findings constitute good baseline data that will be helpful to evaluate the local effectiveness and impact of “vision 2020: the right to sight” programs.

The prevalence of blindness and poor vision found in this study are in keeping with the respective 17% and 18.8% reported by Tielsch et al. [2] who have worked on nursing home residents aged 40 years and above without seeking for their diabetic status. Contrarily, Narendran et al. [14] observed only a 3.5% prevalence of bilateral blindness among 260 self-reported Indian diabetics. This huge discrepancy could be explained by the difference between our study population and that of the above-mentioned authors, as it has been strongly bolstered that blindness is significantly more preponderant in Blacks than in Whites [2]. What’s more, the definitions of blindness and visual impairment we used were not exactly the same as the ones adopted by the previous authors. Indeed, the literature reveals that many factors may contribute to the variation of these prevalence rates, including age (old age especially), duration of diabetes (>10 years), ethnicity, occupation, cognitive function, metabolic control, skill of the examiner and methodology of examination among others [2-5,14,15]. Although women exhibit a tendency to be more blind and visually impaired than men, this has not yet been proven to be statistically significant [2,14]. As a matter of fact, this variation in prevalence rates of blindness and visual impairment highlights the utmost need to standardise the different definitions and classifications regarding visual impairment and blindness, why not by consensually adopting those advocated by ICO [13].

Unsurprisingly, as depicted by some other authors [3,4,14-16], cataract, diabetic retinopathy and glaucoma were the most leading causes of visual impairment and blindness in our patients. The prevalence of cataract we observed (29.7%) is lower than that reported by Rotimi et al. [3] (44.9%), Funatsu et al. [4] (66.7%), Bourne et al. [8] (56%), and by Oye et al. [5] (62.1%). But, it is similar to the 27.1% observed by Tielsch et al. [2], and higher than the 13.1% reported by Roaeid et al. [15] among Libyan diabetics. All these differences could be due to the varying mean age and duration of diabetes among the various study populations.

Meanwhile, the prevalence of diabetic retinopathy we found (17.4%) is lower than what were encountered by Narendran et al. [14] (26.2%), Roaeid et al. [15] (30.6%) and by Funatsu et al. [4] (37%), but comparable to the 17.9% revealed by Rotimi et al. [3] may be because our study populations are of the same race (sub-Saharan Black Africans) [2]. By contrast, our prevalence of diabetic retinopathy is higher than those reported by Tielsch et al. [2] and Bourne et al. [8]: 6.4% and 5% respectively. This difference is mainly explained by the fact that their study populations were not chosen on the basis of a known diabetic status. Although we did not seek for the risk factors for the development of diabetic retinopathy, it is well established that increased age, duration of diabetes (longer than 10 years), methods of diabetic control (HbA1c value), current insulin use, diabetic nephropathy, diabetic neuropathy, hypertension, systolic blood pressure, diastolic blood pressure, and arteriosclerosis obliterans are strongly associated with diabetic retinopathy, which is not the case for the sex [3,4,14]. Therefore, intensive blood glucose control, specifically in the early years of diagnosis (first 5 years), may reduce the risk for the development and progression of retinopathy and cataract. In this regard, early eye examination, preferably at first presentation of elevated blood glucose, is highly recommended [3].

Our prevalence of glaucoma was more than two times comparable to what has been reported by Bella et al. [9] who, after performing a prospective study of the intraocular pressure, fundus and perimetry in 307 Cameroonians aged 20–39 years irrespective of their diabetic status, found a prevalence of 5.8%. Similarly, our prevalence of glaucoma was higher than those witnessed by Merle et al. [6] in Martinique (2.7%), Ramakrishnan et al. [7] in India (2.6%), and Bourne et al. [8] in Thailand (3.8%). This discrepancy could be due to the fact that our study population appears to be older than those of the aforementioned studies. Consequently, our finding could be a true reflection of the prevalence of glaucoma in an aged population at risk especially in our milieu, as it has been clearly pointed out that this prevalence is much lower when working on a younger population of the same milieu [9]. However, the prevalence we obtained could be an overestimate of the real situation, given that we set the threshold for normal intraocular pressure at 20 mmHg instead of 21 mmHg as it was the case in other studies [6-8]. Even though this prevalence seems to be a little bit higher than what it must be, it is nonetheless suggestive of the great importance for a systematic screening of glaucoma in diabetic patients, specifically the aged ones. Tielsch et al. [2] did not show any evidence of an association between diabetes and primary open angle glaucoma, but the relatively high prevalence of neovascular glaucoma (6.67%) we encountered in patients exhibiting proliferative retinopathy in our study is a cause for concern.

Expectedly, we observed in this study that duration of diabetes was associated with glaucoma, cataract, diabetic retinopathy and maculopathy as it has already been pointed out in previous reports [3,4,16]. Poor blood sugar control is very frequent in African countries, leading to complications such as diabetes eye diseases [16]. Interestingly, Rotimi et al. found a low prevalence of diabetic retinopathy and cataract within the first 5 years following the diagnosis of diabetes in a cohort of West African diabetics, suggesting that intensive blood glucose control may reduce the risk of development and progression of retinopathy and cataract in these patients [3]. Similarly to a study aiming at the determination of the prevalence of visual impairment and selected eye diseases among diabetic patients in USA [17], we found that the likelihood for diabetic patients to have cataract and diabetic retinopathy was increased by patients’ age greater than 50 years. Recently, Katte et al. have reported a significant rate of coincident diabetes and hypertension (3.9% in men and 5.0% in women) in a self-selected semi-urban Cameroonian population [18]. Likewise, we found a high frequency of hypertension (39.6%) in our study population and in accordance with previous reports [4,19-21], hypertension increased the risk of glaucoma, cataract and diabetic retinopathy.

A major limitation of this study is the fact that poor vision and blindness due to refractory errors were not analyzed. Not all patients said to have normal or near normal vision had a 10/10 visual acuity. Most of them were prescribed lenses, this suggesting a high prevalence of refractory errors among the estimated 73% of patients with normal or near normal vision. What’s more, we did not perform a systematic evaluation of patients’ visual fields when they were presenting with a high intra-ocular pressure in order to better define glaucoma. Nonetheless, throughout the recruitment, the ophthalmologic examination was carried out by experienced and well trained ophthalmologists so as to have reliable results. Further, the study was undertaken in a hospital environment with adequate and quality-assured equipment.

## Conclusion

Poor vision and blindness are frequent among Cameroonian diabetic patients, and their causes are similar to those reported by various other surveys: cataract, glaucoma, diabetic retinopathy, maculopathy and other less causative ocular affections. Age, sex, duration of diabetes and hypertension are factors that variously impact upon the occurrence of poor vision and blindness among these patients. ‘2020 the right to sight” programs should thereby be focused at tackling and reducing the occurrence as well as the burden of such avoidable ocular affections in our milieu.
